# Brooklyn Dental Society

**Published:** 1873-11

**Authors:** C. P. Crandell


### BROOKLYN DENTAL SOCIETY.

The Brooklyn Dental Society convened for its sixth annual session, Monday evening, Oct. 6, 1873. President Dr. Wm. Jarvie, Jr. in the chair.

The follow ng members were elected officers for the ensuing year.

*President*, Dr. W. T. Shannon ;
*Vice-President*, Dr. L. E. Brockway ;
*Recording Secretary*, Dr. C. P. Crandell ;
*Corresponding Secretary*, Dr. A. N. Chapman ;
*Treasurer*, Dr. F. W. Dolbare ;
*Librarian*, Dr. C. H. Biddle.

The Society is composed of earnest men, thoroughly interested in everything tending to elevate the standard of professional excellence, and enlarge their sphere of usefulness, and a truly noble work is being accomplished as the result of associative effort.

C. P. CRANDELL,

*Rec. Secretary.*

